# Gut Microbiota-Derived Tryptophan Metabolites Alleviate Allergic Asthma Inflammation in Ovalbumin-Induced Mice

**DOI:** 10.3390/foods13091336

**Published:** 2024-04-26

**Authors:** Hongchao Wang, Yuan He, Danting Dang, Yurong Zhao, Jianxin Zhao, Wenwei Lu

**Affiliations:** 1 State Key Laboratory of Food Science and Resources, Jiangnan University, Wuxi 214122, China; hcwang@jiangnan.edu.cn (H.W.); 7230112006@stu.jiangnan.edu.cn (Y.H.); dangdt@163.com (D.D.); 6230111151@stu.jiangnan.edu.cn (Y.Z.); jxzhao@jiangnan.edu.cn (J.Z.); 2 School of Food Science and Technology, Jiangnan University, Wuxi 214122, China; 3 National Engineering Research Center for Functional Food, Jiangnan University, Wuxi 214122, China

**Keywords:** tryptophan metabolites, asthma, gut microbiota, tryptophan metabolism

## Abstract

Asthma is a prevalent respiratory disease. The present study is designed to determine whether gut microbiota-derived tryptophan metabolites alleviate allergic asthma inflammation in ovalbumin (OVA)-induced mice and explore the effect and potential mechanism therein. Asthma model mice were constructed by OVA treatment, and kynurenine (KYN), indole-3-lactic acid (ILA), in-dole-3-carbaldehyde (I3C), and indole acetic acid (IAA) were administered by intraperitoneal injection. The percent survival, weight and asthma symptom score of mice were recorded. The total immunoglobulin E and OVA-specific (s)IgE in the serum and the inflammatory cytokines in the bronchoalveolar lavage fluid (BALF) were detected by the corresponding ELISA kits. The composition of the gut microbiota and tryptophan-targeted metabolism in mouse feces were analyzed using 16S rRNA gene sequencing and targeted metabolomics, respectively. The four tryptophan metabolites improved the percent survival, weight and asthma symptoms of mice, and reduced the inflammatory cells in lung tissues, especially I3C. I3C and IAA significantly (*p* < 0.05) downregulated the levels of OVA-IgE and inflammatory cytokines. KYN was observed to help restore gut microbiota diversity. Additionally, I3C, KYN, and ILA increased the relative abundance of *Anaeroplasma*, *Akkermansia*, and *Ruminococcus_1*, respectively, which were connected with tryptophan metabolic pathways. IAA also enhanced capability of tryptophan metabolism by the gut microbiota, restoring tryptophan metabolism and increasing production of other tryptophan metabolites. These findings suggest that tryptophan metabolites may modulate asthma through the gut microbiota, offering potential benefits for clinical asthma management.

## 1. Introduction

Asthma is a chronic bronchial inflammatory disease involving various cells and cytokines [1]. Recent epidemiological studies indicate a rapid increase in respiratory disease prevalence, with asthma emerging as a major chronic airway disease with significant impacts on patients’ quality of life and socio-economic burdens [2,3]. Current therapies for asthma focus on controlling the pathological condition but do not offer a complete cure, leading to substantial financial burdens for patients and governments [4]. Medicines such as inhaled corticosteroids (ICS), which have anti-inflammatory properties, should be taken daily on a long-term basis to prevent exacerbations. Additionally, therapies used on an as-needed basis, such as short-acting inhaled beta2-agonists (SABAs), are used to relieve asthma symptoms primarily by bronchodilation [5]. Adverse effects due to an overload of drug therapy are recognized as a major contributor to the immense healthcare costs of most asthma patients [6]. The pathogenesis of asthma is complex and not yet fully clear. In recent years, research has found that the immune imbalance between T helper (Th)17 cells and regulatory T (Treg) cells is one of the important reasons for developing asthma [7,8]. Th17 cells are proinflammatory effector T cells, producing interleukin (IL)-17, while Treg cells mediate the anti-inflammatory response, inhibit Th17 cell secretion of IL-17, and suppress Th17 cell differentiation [9]. Th17 and Treg cells have opposing roles, and the immune imbalance between Th17 and Treg cells may lead to dysregulation of the inflammatory response, further leading to asthma [10]. The interaction of cytokines such as interleukin (IL)-4, transforming growth factor-beta 1 (TGF-β1), tumor necrosis factor-alpha (TNF-α), and IL-17 are also implicated in asthma pathogenesis [11,12]. The primary targets for alleviating asthma are immunity and inflammation; the immunological and inflammatory process is the underlying cause of asthma attacks [13,14]. Aryl hydrocarbon receptor (AhR) regulates lung inflammation in asthmatic mice by mediating Th17/Treg cell differentiation [15]. While corticosteroids, long-acting beta agonists, and montelukast can mitigate airway inflammation and relieve bronchospasm, they cannot cure asthma; symptoms often recur upon treatment cessation [16]. Moreover, dexamethasone (DEX), used to treat inflammatory diseases, including asthma, commonly causes gastrointestinal discomfort such as nausea and abdominal pain [17]. Thus, there is an urgent need for effective and safe alternative treatments for allergic asthma.

**Figure 1:**
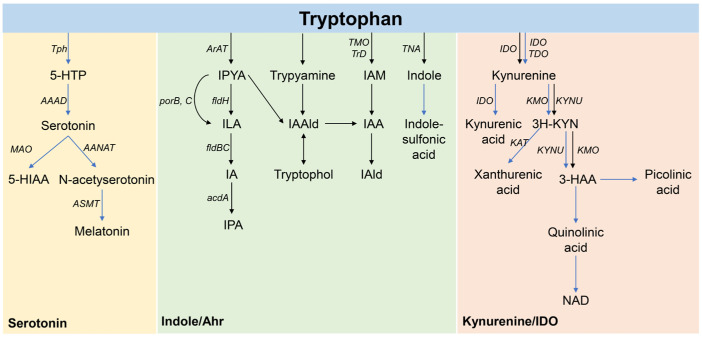
Pathways of tryptophan metabolism; 3-HAA: 3-hydroxyanthranilic acid, 3H-KYN: 3-hydroxykynurenine, 5-HTP: 5-hydroxytryptophan, AAAD: aromatic amino acid decarboxylase, AANAT: aralkylamine N-acetyltransferase, acdA: acv-COA dehvdrogenase, AraT: aromatic amino acid aminotransferase, ASMT: acetylserotonin O-methyltransferase, fldBC: phenyllactate dehydratase, fldH: phenyllactate dehydrogenase, IA: indoleacrylic acid, IAA: indole acetic acid, IAAld: indole-3-acetaldehyde, IAld: indole-3-aldehvde, IAM: indole-3-acetamide, IDO: indoleamine 2,3-dioxygenase, ILA: indole-3-lactic acid, IPA: indole-3-propionic acid, IPYA: indole-3-pyruvate, KAT: kynurenine aminotransferase, KMO: kynurenine 3-monooxygenase, KYNU: kynureninase, MAO: monoamine oxydase, NAD: nicotinamide adenine dinucleotide, porB, C: pyruvate: ferredoxin oxidoreductase B and C, TDO: tryptophan 2,3-dioxygenase, TMO: tryptophan 2-monooxygenase, TNA: tryptophanase, TpH: tryptophan hydroxylase, TrD: tryptophan decarboxylase, blue arrow: human pathway, black arrow: microbial pathway.

Tryptophan is an essential amino acid metabolized in the human gut through three pathways [18] (Figure 1). Firstly, approximately 90% of tryptophan in gut epithelial cells and immune cells degrades into multiple bioactive compounds via tryptophan 2,3-dioxygenase (TDO), indoleamine 2,3-dioxygenase 1 (IDO1), and IDO2. These enzymes result in the accumulation of kynurenine (KYN), known as the KYN pathway. KYN is produced from tryptophan by the catalytic activity of IDO or TDO and is converted into kynurenic acid, 3-hydroxy kynurenine, and anthranilic acid, respectively; 3-hydroxy kynurenine and anthranilic acid are converted into 3-hydroxyanthranilic acid, which finally can be converted into picolinic acid or quinolinic acid through enzymatic or nonenzymatic reactions, respectively [19]. Secondly, about 4–6% of tryptophan in the gut lumen is directly metabolized by the gut microbiota into indole compounds and their derivatives [20]. Thirdly, around 3% of tryptophan in gut chromaffin cells is converted to 5-hydroxytryptamine (5-HT) by tryptophan hydroxylase 1 (TPH1), constituting the 5-HT pathway and producing over 90% of the body’s 5-HT—which is essential for regulating mood, sleep, and other physiological functions.

In recent decades, it has been established that the gut microbiota plays essential roles not only in normal digestion and metabolism but also in promoting immune system development [21]. It is essential to preserve a healthy and stable gut microbiota. An increasing number of studies have demonstrated that inflammatory diseases, such as asthma, inflammatory bowel disease (IBD), and type 2 diabetes, are closely related to dysbiosis of the gut microbiota. Thus, treatments of inflammatory diseases regulating gut microbiota dysbiosis are prospective strategies [22,23]. Asthma induced by ovalbumin (OVA) exacerbates lung tissue inflammation, which appears to be linked to gut microbiota homeostasis [24]. Buendía et al. reported a correlation between increases in *Streptococcus*, *Escherichia coli*, and *Shigella* in the gut and fixed airway obstruction in asthma patients [25]. Furthermore, the severity of asthma has been associated with *Akkermansia* in the gut [26]. These results indicate that microbiota dysbiosis in the gut may lead to the development of asthma, while asthma may lead to changes in gut microbial compositions [27,28]. In addition, metabolites produced by the gut microbiota are helpful for treating asthma. Short chain fatty acids (SCFAs) such as acetate, propionate, and butyrate are produced by bacterial fermentation of dietary fiber [29]. SCFAs have been demonstrated to protect against allergic diseases in mouse models. A study found that SCFAs can mitigate inflammatory cytokines and markers of tissue remodeling in house dust mite (HDM)-induced neutrophilic murine asthma [30]. Meanwhile, it has been reported that SCFA-producing bacteria, such as Bifidobacterium, Lactobacillus and Bacteroides, may regulate inflammatory responses and reduce the risk of asthma [31]. Bile acids, as modulators of the gut microbiota composition and function, can prevent eosinophilic inflammation in primary biliary cirrhosis [29,32].

A growing body of literature suggests that tryptophan catabolites generated by the gut microbiota, including KYN, I3C and IA, are important signaling molecules in microbial communities [33]. The gut microbiota participates in the KYN pathway of tryptophan, modulating tryptophan metabolism and tryptophan metabolite production in the host [19]. Recent studies have found that tryptophan metabolites are assumed to be endogenous AhR ligands [34]. They bind to AhR, activate the immune system, modulate gut homeostasis, and exert anti-inflammatory effects on the systemic circulation [33]. Probiotics that alleviate allergic symptoms can metabolize and produce various gut-derived tryptophan metabolites with potential anti-allergic effects. *Bifidobacterium longum*, which produces indole-3-carbaldehyde (I3C), can effectively alleviate allergic symptoms in mice with atopic dermatitis [35]. *Lactiplantibacillus plantarum* and *Limosilactobacillus reuteri*, which regulate the level of indoleacrylic acid (IA) in the gut, improve food allergies [36]. A study has found that KYN can reduce lung inflammation and attenuate airway hyperreactivity in an OVA-induced asthma mouse model [32]. According to these findings, these results indicate that gut microbiota-derived tryptophan metabolites help alleviate asthma. Hence, targeting tryptophan metabolism by the gut microbiota may offer therapeutic potential for allergic diseases.

Currently, there is no effective preventive strategy for asthma or a known cure, and patients usually have a dependency on pharmacological treatment. Thus, new therapies and therapeutic targets are required for better control of symptoms and exacerbations in asthma patients. Gut microbiota-derived tryptophan metabolites show a great influence on allergic related diseases, while there is limited research on the role of tryptophan metabolism by the gut microbiota in alleviating asthma. Considering the role of gut microbiota-derived tryptophan metabolites in improving asthma, the effects of KYN, indole-3-lactic acid (ILA), I3C, and indole acetic acid (IAA) on OVA-induced asthma were tested in this study. Accordingly, this article mainly studied the inflammatory reactions and pathological characteristics of lungs in asthmatic mice and further explored the underlying mechanisms involving changes in inflammatory cytokines, the gut microbiota and tryptophan metabolism. Ultimately, the experiments verified the connection between tryptophan metabolism and the gut microbiota in alleviating asthma. Therefore, tryptophan metabolism by the gut microbiota may provide a clue for the treatment strategy for asthma patients in future.

## 2. Materials and Methods

### 2.1. Preparation of Tryptophan Metabolite Injections

The powders of KYN, I3C, and IAA were procured from Sigma-Aldrich Co., Ltd. (St. Louis, MO, USA), and ILA was obtained from Aladdin Biochemical Technology Co., Ltd. (Shanghai, China). Polyethylene glycol 300 (PEG 300) was purchased from MedChemExpress Co., Ltd. (Shanghai, China). A total of 80 mg of KYN, ILA, I3C and IAA was dissolved, respectively, in 100 μL of dimethyl sulfoxide (DMSO) (Solarbio Science & Technology, Beijing, China) and then diluted to 10 times by adding a solution (PEG 300/Tween 80 = 8:1). Subsequently, the resulting solution was diluted 10 times with 8 mg/mL saline and mixed well.

### 2.2. Experimental Design

The Ethics Committee of Experimental Animals at Jiangnan University approved the animal testing protocol (JN.No20220915b1281110[318]). Six-week-old female BALB/c mice (specific pathogen-free, SPF), weighing 15–18 g, were procured from Vital River Laboratory Animal Technology Co., Ltd. (Jiaxing, China). All experimental mice were kept in a SPF animal laboratory where ambient environmental conditions (temperature of 20 °C~26 °C, relative humidity of 40%~70%, 12 h light/dark cycle) were maintained. Growth and reproduction feed for mice, SPF level (Co60 irradiation), was provided by Jiangsu Xietong Pharmaceutical Bio-engineering Co., Ltd. (Nanjing, China) and added every 2–3 days. All the mice were free to consume the diet and water. Prior to trials, mice were acclimated to standard laboratory conditions for one week. After acclimation, mice were randomly divided into seven groups (n = 8 per group): control, model, DEX, KYN, ILA, I3C, and IAA groups.

The control group received 50 μL of saline by nasal drip from days 29 to 33 and was treated with 100 μL of saline by intraperitoneal injection from days 29 to 36.

The method of Ma et al. was used to construct an OVA-induced allergic asthma model [37]. OVA was procured from Sigma-Aldrich Co., Ltd. (St. Louis, MO, USA) and aluminum hydroxide was purchased from Sinopharm Chemical Reagent Co., Ltd. (Shanghai, China). All the experimental groups were intraperitoneally injected with 200 μL of the solution (1 mg of OVA dissolved in 1 mL of saline with an equal amount of aluminum hydroxide) on days 8, 15, and 22; mice were treated with 50 μL of OVA (1 mg/mL) by nasal drip from days 29 to 33. The KYN, ILA, I3C, and IAA groups were treated with 100 μL of KYN, ILA, I3C, and IAA (40 mg/kg), respectively, by intraperitoneal injection from days 29 to 36; the DEX group received 100 μL of DEX (2 mg/kg) (Xianju Pharmaceutical, Taizhou, China), and after 2 h of intervention with DEX, mice were administered OVA by nasal drip to elicit an anaphylactic reaction. Mice were sacrificed after the last nasal drip treatment with 50 μL of OVA (1 mg/mL) on day 37. Blood collected from the eyeball was centrifuged at 845× *g* at 4 °C for 15 min to obtain serum. Bronchoalveolar lavage fluid (BALF) was collected according to a previous study [38]. Serum and lung tissues were collected for further analysis.

### 2.3. Weight Measurement and Evaluation of Asthmatic Mice

The weight of the mice was recorded five times at weekly intervals. Body weight percentage (%) was calculated using the following formula:

Percentage of body weight (%) = (current weight)/(8th day weight) × 100%

After OVA challenge, mice were observed for 30 min for upper respiratory symptoms (number of nose scratches and sneezes and amounts of nasal secretions). The mice were then assessed using a superimposed quantitative scoring method, as shown in Table 1 [39]. Record each symptom and add up the scores. A total score greater than five points is considered a successful model. The first stimulation resulted in a death record of nine points; the second stimulation resulted in a death record of eight points; and so on.

### 2.4. Histopathological Changes in Mouse Lung Tissues

The left lung tissues were excised, fixed in 4% paraformaldehyde for at least 24 h, and then embedded in paraffin. Tissue sections were cut and mounted with neutral gum. Hematoxylin and eosin (H&E) staining was performed and images were captured using a microscopy imaging system [40].

### 2.5. Serum Immunoglobulin Assay

Serum was collected and stored at −20 °C. The total level of immunoglobulin E (IgE) in serum was measured using mouse IgE enzyme-linked immunosorbent assay (ELISA) kits (SenBeiJia Biological Technology, Nanjing, China) and the level of ovalbumin-specific immunoglobulin E (OVA-sIgE) was measured using OVA-sIgE ELISA kits (BioLegend, San Diego, CA, USA) according to the manufacturer’s instructions [40].

### 2.6. Measurement of Inflammatory Cytokines in BALF

Mouse IL-6, IL-10, IL-17, and TNF-α ELISA kits (R&D Systems, Minneapolis, MN, USA) were used to measure these cytokines in BALF [41] following the manufacturer’s protocols.

### 2.7. Sequencing the 16S Ribosomal RNA Gene

Fecal samples were collected in sterile centrifuge tubes one day before the mice were sacrificed and were stored at −80 °C for later 16S ribosomal RNA (rRNA) gene analysis according to a previous study [42]. DNA was extracted using fecal DNA extraction kits (MP Biomedicals, Santa Ana, CA, USA) according to the manufacturer’s instructions. PCR amplification of the V3–V4 region of the 16S rRNA gene was performed using the 341F/806R primers. The products were subjected to agarose gel electrophoresis, and the bands were extracted using the QIAquick Gel extraction kits (QIAGEN, Dusseldorf, Germany). After the concentration determination, the pooled samples were sequenced on the MiSeq PE300 Sequencing Platform (Illumina, San Diego, CA, USA). The sequencing data were analyzed using the Qiime2 platform (https://view.qiime2.org/, accessed on 15 October 2023). Pielou’s evenness, Faith’s phylogenetic diversity (Faith_pd) index, and the Shannon index were used to assess alpha diversity, while Constrained Principal Coordinate Analysis (CPCoA) was employed to illustrate beta diversity and was conducted on an online platform (https://www.bic.ac.cn/ImageGP/, accessed on 23 November 2023). Linear discriminant analysis effect size (LEfSe) was performed (https://huttenhower.sph.harvard.edu/galaxy/, accessed on 23 November 2023) to detect differential abundances between groups.

### 2.8. Detection of Tryptophan-Targeted Metabolism in Mouse Feces

Tryptophan-targeted metabolism in mouse feces was analyzed according to a previous study [36]. Mice feces were accurately weighed (50 mg), and then 500 μL of extraction solution (methanol/acetonitrile/water = 2:2:1, pre-cooled at −40 °C, with 0.1% formic acid and an internal standard of isotope labeling) was added. The mixture was vortexed for 30 s and sonicated for 5 min in an ice-water bath (SB 25-12 DTDN, Scientz, Ningbo, China). This ultrasound-assisted extraction was repeated twice. Subsequently, the samples were left to rest at −40 °C for 1 h, followed by centrifugation (13,523× *g*, 15 min, 4 °C). A volume of 320 μL of the supernatant was transferred, dried under nitrogen gas, redissolved in 80 μL of an aqueous solution containing 0.1% formic acid, and then centrifuged at 13,523× *g* at 4 °C for 15 min. The supernatant was analyzed by ultra-high performance liquid chromatography-tandem mass spectrometry (UHPLC-MS/MS) (Thermo Fisher Scientific, Waltham, MA, USA). Chromatographic separation was performed on a column (Waters ACQUITY UPLC HSS T3, 100 mm × 2.10 mm, 1.8 μm, Waters, Milford, MA, USA) maintained at 40 °C. The autosampler temperature was set at 4 °C and the injection volume was 5 μL. Analytes were eluted using a gradient of 0.1% formic acid in water (solvent A) and 0.1% formic acid in acetonitrile (solvent B).

### 2.9. Statistical Analysis

Data are presented as means ± SEMs. Graphs were created and analyzed using GraphPad 8.0 software. One-way ANOVA with Tukey’s multiple comparison test was used for the significance analysis if data were normally distributed; otherwise, Tamhane’s T2 test was applied. An asterisk (*) indicates a significant difference compared with the model group (*p* < 0.05).

## 3. Results

### 3.1. Tryptophan Metabolites Alleviate Changes in Body Weight and Lung Tissues in Allergic Asthma Mice

**Figure 2:**
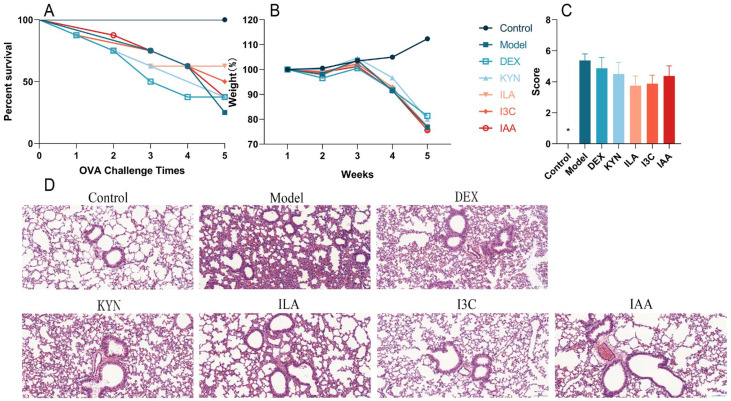
Tryptophan metabolite treatment alleviates allergic symptoms in asthmatic mice. (**A**) Survival rate. (**B**) Body weight. (**C**) Allergy symptom scores. (**D**) Representative images of H&E-stained lung sections, ×150. * Compared with the model group, *p* < 0.05.

Compared with the control group, the survival percentage of the model group gradually decreased with successive OVA challenges. Treatment with tryptophan metabolites, particularly ILA and I3C, improved survival compared with the model group (Figure 2A). Additionally, compared with the control group, the body weights of other groups decreased; however, tryptophan metabolites did not significantly affect body weight compared with the model group (Figure 2B). During the stimulation period, the control group exhibited smooth and shiny fur, gradual weight gain, no scratching behavior, and no decrease in activity. In contrast, the model group displayed disheveled fur, whisker loss due to continuous nose scratching, gradual weight loss, a significant activity reduction, partial eye closure in severe cases, decreased alertness, and unresponsiveness to stimuli. Furthermore, according to the evaluation of asthma symptoms in mice, DEX and tryptophan metabolites alleviated asthma symptoms compared with the model group; however, these improvements were not statistically significant (Figure 2C). The histopathological examination using H&E staining revealed that lung damage in the model group was more severe than that in the control group, with infiltration of inflammatory cells around blood vessels, alveolar collapse, thickening of alveolar walls, and increased inflammatory cells. Compared with the model group, inflammatory cell infiltration around bronchi and alveolar walls decreased in the other groups and the basement membrane appeared thinner; notably, I3C had the most pronounced effect on alleviating these symptoms (Figure 2D). The above results showed that tryptophan metabolites, especially ILA and I3C, could improve allergic symptoms and lung inflammation in asthmatic mice.

### 3.2. Tryptophan Metabolites Regulate IgE and OVA-sIgE Levels in Allergic Asthma Mice

**Figure 3:**
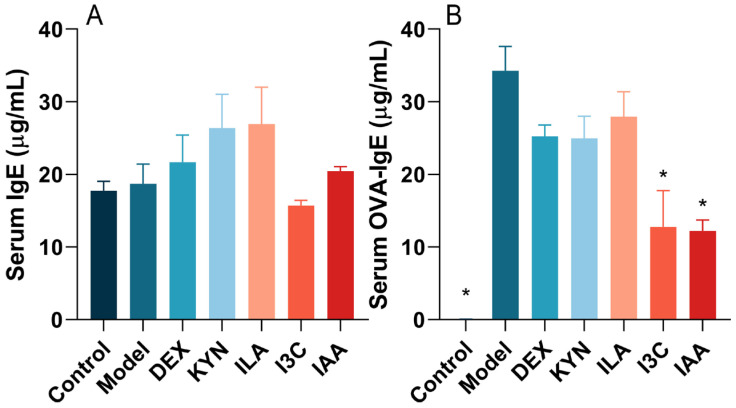
Tryptophan metabolite treatment affects the levels of total immunoglobulin E (IgE) and ovalbumin-specific immunoglobulin E (OVA-sIgE) in the serum of mice. Serum levels of (**A**) total IgE and (**B**) OVA-sIgE. * Compared with the model group, *p* < 0.05.

The experimental results showed that compared with the model group, I3C treatment resulted in a decrease in IgE levels, although not significantly (Figure 3A). As depicted in Figure 3B, the level of OVA-sIgE was upregulated significantly (*p* < 0.05) in the model group. DEX and tryptophan metabolite administration led to a downregulation of OVA-sIgE levels compared to the model group. Notably, the levels of OVA-sIgE in the control, I3C, and IAA groups decreased significantly (*p* < 0.05) compared to the model group.

### 3.3. Tryptophan Metabolites Influence Inflammatory Cytokine Levels in Allergic Asthma Mice

**Figure 4:**
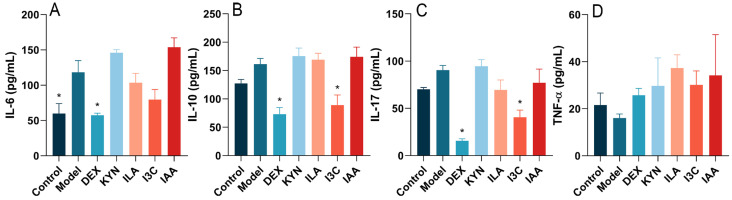
Tryptophan metabolite treatment reduces the levels of inflammatory cytokines in BALF. (**A**) IL-6. (**B**) IL-10. (**C**) IL-17. (**D**) TNF-α. * Compared with the model group, *p* < 0.05.

Figure 4A–C illustrates that, relative to the control group, IL-6 levels increased significantly in the model group, while IL-10 and IL-17 levels also rose but not significantly. Compared with the model group, IL-6 levels in the DEX group decreased significantly (*p* < 0.05) (Figure 4A). DEX and I3C treatments were found to significantly (*p* < 0.05) reduce IL-10 and IL-17 levels compared to the model group (Figure 4B,C). Additionally, TNF-α levels were elevated by DEX and tryptophan metabolites relative to the model group, though these differences were not significant (Figure 4D).

### 3.4. Tryptophan Metabolites Modulate the Gut Microbiota Composition in Allergic Asthma Mice

**Figure 5:**
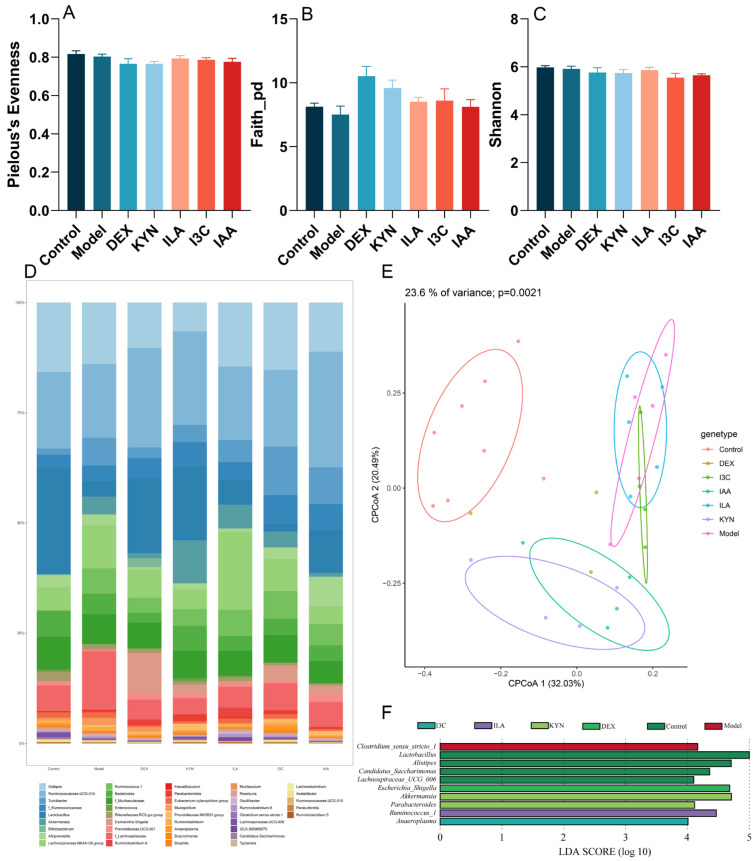
Tryptophan metabolite treatment reshapes the gut microbial composition in asthma model mice. (**A**) Pielou’s index. (**B**) Faith_pd index. (**C**) Shannon index. (**D**) Relative abundance of the gut microbiota at the genus level. (**E**) Constrained Principal Coordinate Analysis (CPCoA). (**F**) Results of linear discriminant analysis effect size (LEfSe).

The analysis revealed that Pielou’s evenness, the Faith_pd index, and the Shannon index were unaffected by tryptophan metabolites or the effect was not significant (Figure 5A–C). Compared with the control group, Pielou’s evenness, the faith_pd index and Shannon index of the model group decreased. After the intervention with tryptophan metabolites, Pielou’s evenness and the Faith_pd index of the experimental groups increased but not significantly. Beta diversity was represented by CPCoA, which showed distinct separation among groups (Figure 5E). The distance between the control and model groups was far. After treatment with tryptophan metabolites, there was a tendency of the KYN group to approach the control group. At the genus level, tryptophan metabolites induced changes in the gut microbiota composition (Figure 5D). As shown in Figure 5F, LDA score results indicated higher relative abundances of *Lactobacillus*, *Alistipes*, *Candidatus_Saccharimonas*, and *Lachnospiraceae_UCG_006* in the control group compared to the other groups. The relative abundances of *Clostridium_sensu_stricto_1*, *Escherichia_Shigella*, *Ruminococcus_1*, and *Anaeroplasma* increased, respectively, in the model, DEX, ILA, and I3C groups. Moreover, the relative abundances of *Akkermansia* and *Parabacteroides* were significantly higher in the KYN group than in the other groups.

### 3.5. Tryptophan Metabolites Influence the Tryptophan Metabolite Content in the Gut Microbiota of Allergic Asthma Mice

**Figure 6:**
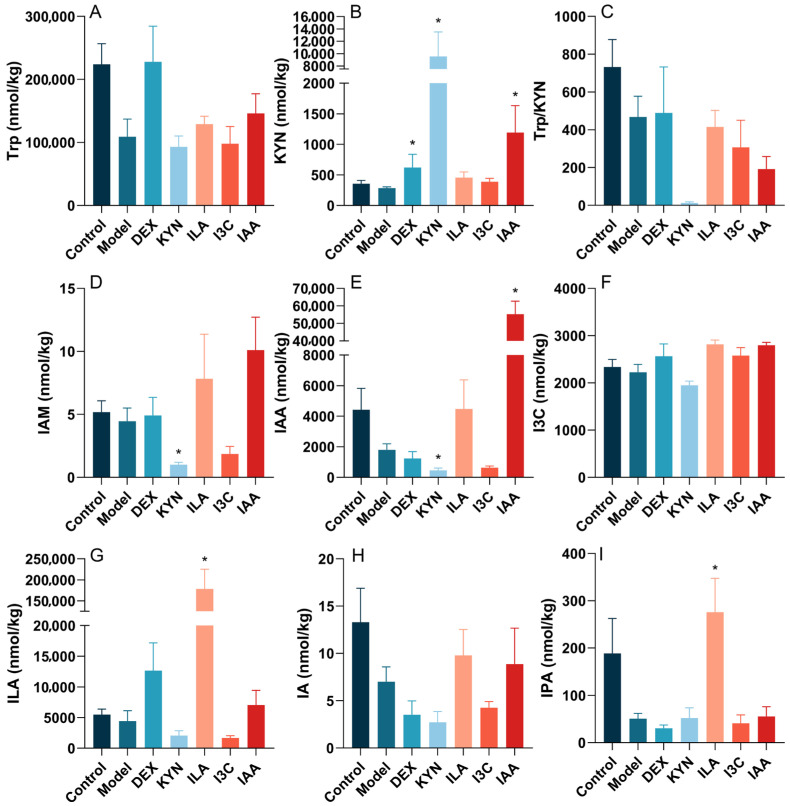
Tryptophan metabolite treatment influences the final concentration of tryptophan (Trp) metabolites in the gut microbiota of mice. (**A**) Trp. (**B**) Kynurenine (KYN). (**C**) Trp/KYN. (**D**) Indole-3-acetamide (IAM). (**E**) Indole acetic acid (IAA). (**F**) Indole-3-carboxaldehyde (I3C). (**G**) Indole-3-lactic acid (ILA). (**H**) Indoleacrylic acid (IA). (**I**) 3-Indolepropionic acid (IPA). * Compared with the model group, *p* < 0.05.

In order to study the relationship between pure tryptophan metabolites and fecal tryptophan metabolites, the absolute quantification of target metabolites in mouse feces was analyzed, and results are shown in Figure 6. The levels of tryptophan (Trp), KYN, Trp/KYN ratio, indole-3-acetamide (IAM), IAA, I3C, ILA, IA, and 3-indolepropionic acid (IPA) decreased in the model group compared to the control group, albeit not significantly. Treatment with DEX, ILA, and IAA elevated Trp levels compared to the model group, whereas KYN and I3C treatment reduced Trp levels; however, these changes were not significant (Figure 5A). DEX, KYN, and IAA treatments significantly (*p* < 0.05) increased KYN levels compared to the model group (Figure 6B). The Trp/KYN ratio decreased in the experimental groups relative to the control group, but not significantly (Figure 6C). KYN treatment notably (*p* < 0.05) reduced IAM and IAA levels (Figure 6D,E). The level of I3C was lower in the KYN group compared to the model group (Figure 6F). ILA treatment significantly (*p* < 0.05) increased ILA levels (Figure 6G). The level of IA in the ILA and IAA groups rose compared to the model group, but not significantly (Figure 6H). Finally, ILA treatment significantly (*p* < 0.05) enhanced IPA levels relative to the model group (Figure 6I).

## 4. Discussion

The pathogenesis of asthma has been linked to the gut microbiota [13], which plays a pivotal role in gut tryptophan metabolism [43]. Consequently, modulating tryptophan metabolism by the gut microbiota could be an effective strategy for improving or alleviating allergic symptoms.

Asthma often leads to severe weight loss and recurrent symptoms such as sneezing, wheezing, shortness of breath, and coughing in mice [44]. In this experiment, compared to the control group, the percent survival and weight of the model group decreased progressively with increased OVA stimulation, and the asthma symptom score increased. However, ILA and I3C reduced mortality and alleviated asthma symptoms in asthmatic mice. Asthma has been shown to be closely associated with lung function and inflammation [45]. It has been found that the number of inflammatory cells increases and they infiltrate the airways, and the connective tissue becomes thickened in the lungs of asthma patients [46]. The model group exhibited thicker bronchi, deformed lung tissues, and inflammatory cell infiltration compared to the control group, consistent with previous research [47]. Notably, gut microbiota-derived tryptophan metabolites reduced inflammatory cells, and I3C especially reversed these pathological conditions, indicating that gut microbiota-derived tryptophan metabolites could relieve lung tissue inflammation in asthmatic mice.

Asthma is considered a canonical type 2 disease, with frequent observations of atopy, eosinophilia, and elevated allergen-specific IgE levels [48]. Mechanistically, asthma is an immunoreactive disease primarily caused by an imbalance of the Th1/Th2 ratio and dysfunction [49]. Additionally, it is commonly observed that Th1 cell levels decrease and Th2 cell levels increase, along with hyperactive Th2 cells [50]. Th2 cells, which predominate in allergic reactions and asthma, are the primary source of the type 2 cytokines IL-4, IL-5, and IL-13 [48]. IgE, a kind of immunoglobulin, mainly mediates immediate allergy and participates in the airway inflammation and airway remodeling of obstructive airway diseases [51]. When IgE binds to mast cells and basophils, it will trigger complex signaling cascades that release inflammatory and vasoactive mediators such as histamine, leukotrienes, and vasopressin. These mediators will cause clinical responses that ultimately lead to asthma [52]. In this study, the synthesis and secretion of OVA-sIgE were enhanced in the OVA-induced asthma mouse model, likely due to decreased Th1 cell levels and increased Th2 cell levels. Meanwhile, the OVA-sIgE level was highest in the model group, aligning with previously reported results [53]. Moreover, tryptophan metabolites, particularly I3C and IAA, reduced OVA-sIgE levels. These findings suggest that gut microbiota-derived tryptophan metabolites can alleviate asthma.

Allergic asthma may result from an imbalance in Th1/Th2 cells, while worsening of the condition is linked to an imbalance in Th17/Treg cells [54]. The severity and control of asthma may be connected with increased Th2 and Th17 cell responses and decreased Treg cells in patients [54]. IL-6 is a cytokine synthesized and secreted by various cell types, including Th2 cells, and can be both proinflammatory and anti-inflammatory [55]. TNF-α is considered a pro-inflammatory cytokine associated primarily with Th1-type reactions involved in allergic respiratory responses [56,57]. In this experiment, IL-6 levels were upregulated in OVA-induced allergic asthma mice, indicating high levels and overactive Th2 cytokines, leading to airway hyperreactivity and asthma [58]. Compared to the model group, ILA and I3C reduced IL-6 levels and increased TNF-α levels, although not significantly. These results suggest that ILA and I3C may promote a shift from Th2 to Th1 cells, further helping alleviate asthma. Th17 cells produce IL-17 and enhance Th2 cell-mediated eosinophilic airway inflammation, which plays a significant role in autoimmune and inflammatory diseases [59,60]. Treg cells release cytokines such as IL-10 to control antigen-specific inflammation and suppress overactive Th2 responses [54]. Importantly, Treg cells can also inhibit Th17 cell secretion of IL-17 and suppress Th17 cell differentiation [61]. Previous studies indicate that the imbalance of Th17/Treg cells participates in the immune milieu of asthma [10,62]. The findings indicate that I3C significantly (*p* < 0.05) downregulated IL-10 and IL-17 levels compared to OVA treatment, indicating that I3C helps regulate the balanced pathway of Th17/Treg cells to alleviate asthma’s inflammatory symptoms. In addition, research reported that I3C may bind AhR to inhibit RORγt Treg cell growth and promote in increase in Gata3 Treg cells, thereby alleviating inflammation [63,64]. Thus, the above results suggest that gut microbiota-derived tryptophan metabolites may alleviate asthma via modulating the balance of Th1/Th2 cells and Th17/Treg cells.

Dysbiosis of the gut microbiota has been recognized as a potential diagnostic tool for asthma [63]. Emerging evidence indicates dysbiosis in patients with asthma, as well as in mouse models [64,65]. In this study, it was found that Pielou’s evenness, the Faith_pd index and the Shannon index of the model group decreased, indicating that the number, evenness, and richness of the gut microbiota in asthmatic mice decreased and the gut microbiota became disordered, which aligns with previous research [66]. However, treatment with tryptophan metabolites increased Pielou’s evenness and the Faith_pd index of the experimental groups, indicating that tryptophan metabolites help improve the richness and evenness of the gut microbiota, but probably reduce the diversity. The beta diversity analysis showed that OVA significantly alters the composition of the gut microbiota, but KYN helps restore the diversity of the gut microbiota, indicating that KYN may regulate intestinal homeostasis by reshaping or restoring the structure of the gut microbiota. A study discovered that *Ruminococcus*, an anti-inflammatory bacterium, was reduced in stool samples from patients with hepatitis B virus-associated hepatocellular carcinoma [67]. Moreover, previous research found that *Ruminococcus* can convert hydrogen and carbon dioxide to acetic acid, which is used by other butyrate-producing microorganisms [68]. Notably, the present study revealed that ILA could address intestinal microecological imbalances in asthmatic mice by increasing the abundance of the key bacterium *Ruminococcus_1* at the genus level [69]. It was found that the relative abundance of *Lactobacillus* was downregulated by OVA, while it was upregulated by tryptophan metabolites. Studies have found that *Lactobacillus* supplementation can temporarily change the delayed development of the gut microbiota in high-risk infants with asthma [70], indicating that gut microbiota-derived tryptophan metabolites can regulate gut microbiota disorders by increasing the relative abundance of *Lactobacillus*, and further alleviate asthma. Additionally, this study suggests that an intraperitoneal injection of KYN resulted in *Akkermansia* becoming a dominant microbe in mice, consistent with reports that the relative abundance of *Akkermansia muciniphila* is reduced in patients with severe asthma [26]. Tryptophan metabolites have been shown to promote the relative abundance of *Akkermansia* [71], and results in this study support this finding. *Akkermansia* is known to play a beneficial role in the gut microbiota, reducing lung eosinophils and airway hyperreactivity, and regulating inflammatory cytokines through secreted metabolites, which help alleviate asthma [26,72]. These findings suggest that tryptophan metabolites also can upregulate the beneficial microbes *Parabacteroides*, *Akkermansia*, *Ruminococcus_1*, and *Anaeroplasma*, many of which participate in bacterial tryptophan metabolism, to activate AhR expression and further attenuate inflammatory responses in mice with asthma [73].

Through a targeted metabolomics analysis, this study observed that, after modeling, the levels of KYN, IAM, IAA, I3C, ILA, IA, and IPA decreased in mouse feces. Asthma reduced the levels of various intestinal tryptophan metabolites in mouse feces, weaking the capability of tryptophan metabolism by the gut microbiota. Importantly, it has been reported that the activity of IDO-1 can be estimated by Kyn/Trp concentrations [67]. The level of KYN/Trp is downregulated by OVA, indicating that asthma may decrease IDO-1 activity, altering the KYN pathway and further disrupting tryptophan metabolism by the gut microbiota. Tryptophan metabolism disrupted by asthma can interfere with host–gut symbiotic bacteria interactions, affecting Treg cell homeostasis [74]. Additionally, the decreases in Treg cell function and quantity are obvious manifestations of immune disorders in asthma patients and an important factor in the pathogenesis of asthma [8]. It has been reported that tryptophan metabolites help increase Treg cells, and decrease airway inflammation and inflammatory cytokines in a mouse model of OVA-induced allergic asthma [75]. Intervention with these four tryptophan metabolites appeared to restore target gut-derived tryptophan metabolites and intestinal homeostasis in the asthmatic mice. In line with this, it was found that intervention with these four tryptophan metabolites substantially enhanced the concentrations of tryptophan and its metabolites, including KYN, ILA, I3C, IAA, IA, IPA and IAM, in asthmatic mice. Tryptophan metabolites, particularly indole derivatives from dietary sources and the gut microbiota, act as ligands for AhR, a potent modulator of immunity [76]. Previous studies show that tryptophan metabolites suppress inflammatory responses in macrophages, and AhR signaling has an important role in the function of macrophages [77]. AhR downregulates the production of the proinflammatory cytokine IL-6 by suppressing histamine production in macrophages [71]. The binding of these metabolites to AhR contributes to intestinal permeability, inflammation regulation, and host immunity [78]. The AhR pathway is complex, as it is potently expressed in the most immune cells involved in the pathophysiology of asthma [79]. The activation of AhR by tryptophan metabolites regulates the expression of Th17 and Treg cells and their corresponding pro-inflammatory and anti-inflammatory factors in mouse lungs [8]. Thus, these four pure tryptophan metabolites, especially IAA, could promote microbial tryptophan metabolism and the production of tryptophan metabolites, further activating and binding to AhR, thereby improving intestinal barrier integrity, and ultimately alleviating asthma.

This study had some potential limitations. Firstly, mouse models are used for almost all types of allergic disease, but no single model accurately models all the features of asthma [80]. Asthma is a complex disease. Only one mouse model was used in this experiment. Considering the different pathological characteristics of asthma, different types of allergens are supposed to be used construct different models of asthmatic mice to explore the effects of tryptophan metabolites on patients with different asthma characteristics. Secondly, there are differences in the asthma symptoms of humans and mice. This experiment is limited to a mouse model, and population experiments were not conducted. Thus, the study should be complemented by findings in humans and subsequent clinical trials on alleviating asthma should be conducted. Fourthly, future studies may need to further detect Treg and Th17 cells according to the method of Hu et al. [75]. Fifthly, it has been confirmed that the gut microbiota participates in tryptophan metabolism and alleviates asthma, but the specific mechanism is unclear. Subsequent screening of the gut microbiota that can utilize tryptophan metabolites may be necessary. Lastly, this study only explored four gut microbiota-derived tryptophan metabolites, and the effectiveness of other gut microbiota-derived tryptophan metabolites, such as IPA, IA, indole aldehyde (IAld), and others, remains to be explored.

## 5. Conclusions

In conclusion, this study investigated the effects of four gut microbiota-derived tryptophan metabolites—KYN, ILA, I3C, and IAA—on asthma. It was found that I3C relieved lung inflammation, significantly reduced OVA-sIgE levels in mouse serum, and downregulated inflammatory cytokines such as IL-10 and IL-17 in BALF. KYN helped restore the gut microbiota diversity, further alleviating OVA-induced allergic asthma inflammation through the gut microbiota. Additionally, these four tryptophan metabolites, particularly IAA, could enhance tryptophan metabolism in the gut and increase the production of tryptophan metabolites, which can further bind to AhR to alleviate asthma. In summary, gut microbiota-derived tryptophan metabolites alleviate allergic asthma inflammation in OVA-induced mice. The present study provides novel insights into the mechanism of gut microbiota-derived tryptophan metabolites in the treatment of asthma and contributes to the future development of drugs related to tryptophan metabolites for the treatment of asthma. Of course, the study provides a new therapeutic direction to treat asthma, focusing on the gut microbiota, and provides a foundation for future research on the role of tryptophan metabolism by the gut microbiota in alleviating asthma.

## Figures and Tables

**Table 1:** The evaluation of asthma symptoms in mice.

Score	1 (Mild)	2 (Moderate)	3 (Severe)
Nasal itching	Slight scratching of the nose with the front paws (<10 times)	Scratching the nose (between slight scratching and rubbing around)	Severe scratching of the nose (rubbing around)
Sneeze number	1–3	4–10	≥11
Runny nose	Flowing to the front nostril	Flowing beyond the front nostrils	Flowing all over the face

## Data Availability

The original contributions presented in the study are included in the article; further inquiries can be directed to the corresponding author.

## Abbreviations

AhR, aryl hydrocarbon receptor; BALF, bronchoalveolar lavage fluid; CPCoA, Constrained Principal Coordinate Analysis; DEX, dexamethasone; DMSO, dimethyl sulfoxide; ELISA, enzyme-linked immunosorbent assay; Faith_pd, Faith’s phylogenetic diversity; HDM, house dust mite; H&E, hematoxylin and eosin; IAA, indole acetic acid; IAld, indole aldehyde; IAM, indole-3-acetamide; IBD, inflammatory bowel disease; ICS, inhaled corticosteroids; IDO1, indoleamine 2,3-dioxygenase 1; IgE, immunoglobulin E; IL, interleukin; ILA, indole-3-lactic acid; IPA, 3-indolepropionic acid; I3C, indole-3-carbaldehyde; KYN, kynurenine; LEfSe, linear discriminant analysis effect size; OVA, ovalbumin; OVA-sIgE, ovalbumin-specific immunoglobulin E; PEG 300, polyethylene glycol 300; rRNA, ribosomal RNA; SCFAs, short chain fatty acids; SPF, specific pathogen-free; SABAs, short-acting inhaled beta2-agonists; TDO, tryptophan 2,3-dioxygenase; TGF-β1, transforming growth factor-beta 1; Th, T helper cell; TNF-α, tumor necrosis factor-alpha; TPH1, tryptophan hydroxylase 1; Treg, regulatory T; Trp, tryptophan; UHPLC-MS/MS, ultra-high performance liquid chromatography–tandem mass spectrometry; 5-HT, 5-hydroxytryptamine.
